# Subjective cognitive decline as a predictor of future cognitive decline: a systematic review

**DOI:** 10.1590/1980-57642020dn14-030007

**Published:** 2020

**Authors:** Vladimir Anatolevich Parfenov, Vladimir Vladimirovich Zakharov, Anastasia Romanovna Kabaeva, Natalya Vasilyevna Vakhnina

**Affiliations:** 1 Sechenov First Moscow State Medical University - Moscow, Moskva, Russian Federation.

**Keywords:** cognition, dementia, cognitive dysfunction, aging, Alzheimer disease., cognição, demência, comprometimento cognitivo leve, envelhecimento, doença de Alzheimer

## Abstract

Over 44 million people suffer from dementia around the world. Researchers estimated that there will be 48.1 million people with dementia by 2020 and 90.3 million by 2040. In addition to dementia, mild cognitive impairment (MCI) and subjective cognitive decline (SCD) relate to cognitive impairment. It has been established that MCI precedes dementia, however the significance of SCD is still unclear. Recent studies suggest that SCD could be a risk factor for objective cognitive impairment. SCD is defined as а self-estimated decline in cognitive capacity in comparison to an individual’s previous level of functioning, which cannot be determined by neuropsychological tests.

**Objectives:**

To perform a systematic review of prospective longitudinal cohort studies that assessed the risk of MCI and dementia among people with SCD.

**Methods:**

A search was carried out for all available peer-reviewed articles in English related to SCD in PubMed and PsychINFO databases from database initiation through January 2020. The keywords used for the search were ‘subjective cognitive (or memory) impairment (or decline or complaints)’. Three authors separately determined the inclusion or exclusion of all articles retrieved for full-text evaluation.

**Results:**

The chance of progression to dementia in the SCD group was 2.17 (95% confidence interval [95%CI] 1.53‒3.07; p<0.05) compared to normal aging. Furthermore, the SCD group was 2.15 times more likely to progress to MCI than the group without SCD (95%CI 1.39‒3.30; p=0.005).

**Conclusions:**

SCD might precede cognitive impairment, however, more detailed longitudinal studies should be conducted.

## RESUMO.

Mais de 44 milhões de pessoas sofrem de demência em todo o mundo. Pesquisadores estimam que haverá 48,1 milhões de pessoas com demência até 2020 e 90,3 milhões até 2040. Além da demência, o comprometimento cognitivo leve (CCL) e o declínio cognitivo subjetivo (DCS) estão relacionados ao comprometimento cognitivo. Foi estabelecido que o CCL precede a demência, porém a significância do DCS ainda não é clara. Estudos recentes sugerem que o DCS pode ser um fator de risco para comprometimento cognitivo objetivo. DCS é definido como um declínio auto-estimado da capacidade cognitiva em comparação com o nível anterior de funcionamento do indivíduo, que não pode ser determinado por testes neuropsicológicos.

**Objetivos:**

Realizar uma revisão sistemática de estudos prospectivos de coorte longitudinal que avaliaram o risco de CCL e demência entre pessoas com DCS.

**Métodos:**

Foram pesquisados todos os artigos revisados por pares disponíveis em inglês relacionados com DCS nos bancos de dados PubMed e PsychINFO desde o início do banco de dados até janeiro de 2020. As palavras-chave utilizadas para a pesquisa foram “declínio cognitivo (ou de memória) subjetivo (ou comprometimento ou queixas)”. Três autores determinaram separadamente a inclusão ou exclusão de todos os artigos que foram recuperados para avaliação em texto completo.

**Resultados:**

A chance de progressão para demência no grupo com DCS foi de 2,17 (intervalo de confiança de 95% [IC95%] 1,53‒3,07; p<0,05) em comparação ao envelhecimento normal. Além disso, o grupo com DCS teve 2,15 vezes mais chances de progredir para CCL do que o grupo sem DCS (IC95% 1,39‒3,30; p=0,005).

**Conclusões:**

o DCS pode preceder o comprometimento cognitivo, no entanto, estudos longitudinais mais detalhados devem ser realizados.

## INTRODUCTION

Over 44 million people worldwide have dementia.^1^ Researchers estimated that there will be 48.1 million people with dementia by 2020 and 90.3 million by 2040.^1^ Cognitive impairment is a very common cause of disability in the elderly.

It is well known that in the most common dementing disorders, *e.g*. in Alzheimer disease, clinical symptoms develop only after a long period of silent progressive brain damage. It has been established that mild cognitive impairment (MCI) precedes dementia; however, the significance of subjective cognitive decline (SCD) is still unclear.

Recent studies suggest that SCD could be the earliest symptom of the dementing disorder.^2^ ^,^ ^3^ ^,^ ^4^ ^,^ ^5^ Reisberg et al., in 1982 and 1986, assumed that subjective complaints constitute the second stage of dementia according to the Global Deterioration Scale and precede objective cognitive decline.^6^ ^,^ ^7^

Considering SCD as a preclinical stage of a dementing disorder, a working group of SCD researchers published key definitions and a conceptual framework for research on SCD.^8^ SCD was defined as a self-estimated decline in cognitive capacity compared to and individual’s previous level of functioning, which cannot be determined by neuropsychological tests. This condition was thought to occur when mild neuronal damage can be compensated functionally.

Several studies have shown that the prevalence of SCD is relatively high in a common elderly population.^9^ ^,^ ^10^ ^,^ ^11^ SCD could precede cognitive impairments of different etiologies (Alzheimer disease, vascular dementia, Lewy body dementia). However, SCD is an unspecific symptom and can be a result of the normal aging process or can be caused by conditions other than cognitive impairment, such as psychiatric disorders (depression, anxiety, and neuroticism), sleep problems, medication, or substance abuse.^8^ ^,^ ^11^ ^,^ ^12^ ^,^ ^13^

The main goal of this study was to establish relations between SCD and objective cognitive impairment and examin the ability of SCD to predict MCI or dementia.

## METHODS

We performed a detailed review of all available peer-reviewed articles available in English that referred to SCD in PubMed and PsychINFO databases from the beginning of the database through January 2020. The keywords used for the search were ‘subjective cognitive (or memory) impairment (or decline or complaints)’. After acquiring the initial search results, the titles and abstracts of the articles were evaluated for suitability against the selection criteria. Full-text articles were then retrieved and assessed for inclusion. Three authors separately determined the inclusion or exclusion of all articles retrieved for full-text evaluation.

The quality of the studies was assessed by 2 reviewer authors using the Newcastle-Ottawa Scale (NOS), as recommended by the Cochrane Non-Randomized Studies Methods Working Group. Inclusion criteria were:

- Prospective longitudinal cohort studies published from January 2006 to January 2020;
- Follow-up period of 12 months and longer;
- Presence of a control group.

Studies where participants had baseline objective cognitive decline were excluded, as well as studies that included participants with SCD and other coexisting diseases, which could be a cause of memory complaints.

Data extraction was performed using a designed form by two authors. The information was collected about study details (year of the study, follow-up period, settings, method of SCD assessment, method of cognitive function assessment, MCI and dementia criteria), and demographic features (number of participants in SCD and control groups, mean age, percentage of females, mean Mini-Mental State Examination [MMSE] score in the SCD group). Results of the study (number of cases from both groups that converted to MCI and dementia) were accurately extracted. The reviewers encountered disagreement such as differences in selection of time points, control groups, scales, and whether to include a study in the review. Disagreements about data extraction were solved by consensus or by the decision of a third reviewer. In case of possible duplications, only one main study was included.

SCD was defined by the criteria used in each study. MCI was defined using Petersen criteria.^14^ The amount of dementia conversion cases was determined by the criteria used in each study.

Three main types of calculations were performed. First, cumulative conversion rates of SCD to dementia or to MCI were calculated. This parameter shows how many participants with SCD develop objective cognitive impairment during follow-up. Secondly, cumulative conversion rates of control for dementia or MCI were calculated. Finally, the relative risks of dementia or MCI were calculated. This statistical parameter indicates whether participants in the SCD group are more likely to develop dementia or MCI than participants without SCD.

Besides cumulative conversion rates, annual conversion rates were calculated for all kinds of outcomes. The annual conversion rate was calculated by dividing the number of subjects who progressed by the follow-up period of each subject.

A weighted proportion analysis (DerSimonian-Laird model) was used in this study. The data set’s heterogeneity was measured using the I^2^ parameter. Publication bias was assessed via Egger’s and Egger-Harbord’s tests and funnel plot inspection.

Due to the possible heterogeneity of the results, the relative risk was calculated for each dementia criteria that were used in the studies. The Knapp and Hartung adjustment was also used to account for uncertainty in the assessment of residual heterogeneity.

The statistical software StatsDirect was used to create the figures.

## RESULTS

**Figure 1.**
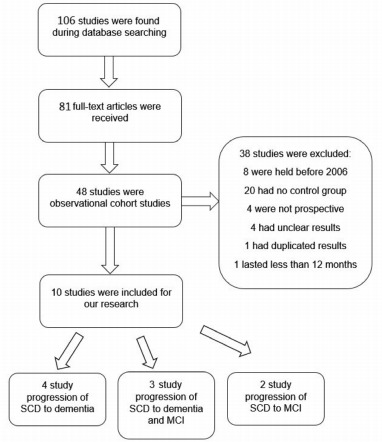
Study selection scheme.

SCD: subjective cognitive decline; MCI: mild cognitive impairment.

A total of 106 potentially eligible articles from keyword search were identified, which referred to the association between SCD and objective cognitive decline. Twenty-five articles were not available in the full version. Eighty-one full-text articles were retrieved. Seventy-one studies were excluded for not meeting the study selection criteria. In the excluded articles, 33 reports were not observational cohort studies; 8 studies were held before 2006; 20 studies had no control group; 4 studies were not prospective; 4 studies had unclear results; 1 study duplicated results of the included study; 1 study had follow-up time less than 12 months. As a result, 10 articles were included in our systematic review.^15^ ^,^ ^16^ ^,^ ^17^ ^,^ ^18^ ^,^ ^19^ ^,^ ^20^ ^,^ ^21^ ^,^ ^22^ ^,^ ^23^ ^,^ ^24^ Of these 10 articles, 4 studies considered SCD progression to dementia, 3 studies evaluated the conversion of SCD to MCI or dementia, and 2 studies analyzed the association between SCD and MCI. The stages of study selection are presented in Figure 1.

A total of 8,128 people participated in the included studies. SCD groups included 4,331 individuals and control groups included 3,797 ones. The mean age of participants with SCD was 73.68±6.26 years, and the mean percentage of females in the studies was 58.51%. The mean age of the control group subjects was 72.92±6.07 years. The mean baseline MMSE of individuals with and without SCD was 28.5±1.8 and 28.7±1.4, respectively. The mean education level of SCD participants from 5 studies was 13.78±3.02 years. Participants from the studies were recruited mostly from the community, but there were also participants from general practice, memory clinics. Healthy controls were recruited mostly from the community, general practice. The mean follow-up time in dementia studies was 5.27 years, and in MCI studies, 4.91 years. Of the different criteria commonly used for dementia diagnosis, the authors of the included studies used the Diagnostic and Statistical Manual of Mental Disorders 4^th^ Edition (DSM-IV), the International Classification of Diseases, 10^th^ Edition (ICD-10), the Clinical Dementia Rating (CDR), the Mini-Mental State Examination (MMSE), Neurological and Communicative Disorders and Stroke and The Alzheimer’s Disease and Related Disorders Association Criteria (NINCDS-ADRDA), Brief Cognitive Rating Scale (BCRS). For MCI diagnosis, the authors applied Petersen, CDR, and NIA-AA criteria developed by the working group in MCI of the European Alzheimer’s Disease Consortium. More detailed characteristics of the included studies are presented in Table 1.

**Table 1.** Features of the included studies.

Study	Number of SCD group	% F	Mean age of SCD group (years)	Number of control group	Mean age of control group (years)	Settings	Method of SCD assessment	Follow-up time (years)	Average MMSE in SCD group	Average MMSE in control group	Education among SCD group (years)	Dementia criteria	MCI criteria
Ferreira^15^	81	51	72.8±7.1	68	72.7±7.2	Community	Do you have difficulties with your memory?	7.5	28.6±1.3	29.0±1.1	NS	NINCDS-ADRA	Petersen
Luck^16^	162	73.8	82.3±4.4	281	82.3±4.4	Community	Do you have problems with your memory?	8	26.8±3.2	27.7±2.4	NS	DSM-IV	
Jessen^17^	1061	58.3	79.8±3.5	863	79.7±3.5	GP	Do you feel like your memory is becoming worse?	6	NS	NS	NS	DSM-IV, ICD-10	
Mol^18^	94	NS	66.4±7.3	297	66.4±7.3	Community	Do you consider yourself to be forgetful?	6	28.1±2.0	28.3±1.6	NS	MMSE <24	
Reisberg^19^	166	65	67.5±8.9	47	64.1±8.9	Community	GDS	6.8	29.0±1.2	29.6±0.8	15.6±2.6	MMSE, BCRS	
Fernandez-Blazquez^20^	423	67	73.8	185	74.2±4.0	Community	Are you easily distracted? Do you get lost in familiar surroundings or have trouble founding your way when driving? Do you often forget recent information or events? Do you often forget autobiographically information? Do you have trouble recognizing objects or faces? Do you have word-finding difficulties for people's names or common words? Do you understand simple verbal and written instructions? Do you have difficulty driving, managing finances or planning daily activities? Do you have difficulty sequencing movements? SCD-scale	1.1	28.6	28.6±1.6	NS		NIA-AA
Donovan^21^	56	56.3	76.4±6.5	283	69.6±9.8	Community, memory clinics	Not stated	2.43	29.0±1.4	29.2±1.0	17.1±2.3	CDR	CDR
Tsutsumimoto^22^	2006	46.8	71.6±5.3	919	71.2±4.9	Community	CAMDEX questionnaire; Do you have any difficulty with your memory? Do you forget where you have left things more than you used to? Do you forget the names of close friends or relatives? Do other people find you forgetful?	2	NS	NS	11.5±2.5	ICD-10	
Nunes^23^	15	66.7	65.9±7.7	11	69.6±5.5	Memory clinic	SMC scale	3.4	29.2±0.8	29.0±0.9	10.4±5.0	DSM-IV TR	Criteria by the Working Group in MCI of the European Alzheimer's Disease Consortium
van Harten^24^	267	50.2	80.3±5.6	843	79.4±5.2	Community	Adapted Blessed scale, Everyday Cognition Scale, “Are you concerned you have a memory or thinking problem?”	6.7	NS	NS	14.3±2.7		Petersen

SCD: subjective cognitive decline; MMSE: Mini-Mental State Examination; MCI: mild cognitive impairment; NINCDS-ADRA: Neurological and Communicative Disorders and Stroke and the Alzheimer’s Disease and Related Disorders Association Criteria; NS: not stated; DSM-IV: Diagnostic and Statistical Manual of Mental Disorders; GP: general practice registry based longitudinal study; BCRS: Brief Cognitive Rating Scale; NIA-AA: National Institute on Aging and Alzheimer’s Association; CDR: clinical dementia rating; CAMDEX: Cambridge Examination of Mental Disorders of the Elderly; ICD-10: International Statistical Classification of Diseases and Related Health Problems 10^th^ Revision; DSM-IV TR: Diagnostic and Statistical Manual of Mental Disorders 4^th^ Edition, Text Revision.

### Analysis of subjective cognitive decline progression to dementia

The following results were obtained from the analysis of 8 studies.

**Figure 2.**
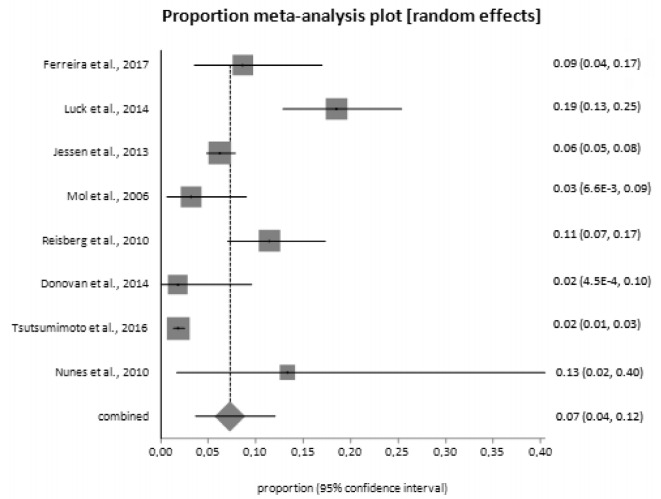
Cumulative conversion rate of subjective cognitive decline to dementia.

Pooled cumulative conversion rate of SCD for dementia was 7.23% (95%CI 3.64‒12.04) (Figure 2). Heterogeneity was high (I^2^=93.30%; 95%CI 89.70‒95.20) and there was some evidence of bias (Harbord-Egger bias=3.89; p=0.16). Annual conversion rate for dementia among SCD participants was 1.12% (95%CI 0.81‒1.49). Heterogeneity was not high (I^2^=0.00%; 95%CI 0.00‒56.30).

Cumulative conversion rate for dementia in the control group was 2.02% (95%CI 0.44‒4.73). Heterogeneity was high (I^2^=92.10%; 95%CI 87.40‒94.50) and there was some evidence of bias (Harbord-Egger bias=0.60; p=0.87). Annual conversion rate among control group participants was 0.45% (95%CI 0.21‒0.76).

**Figure 3.**
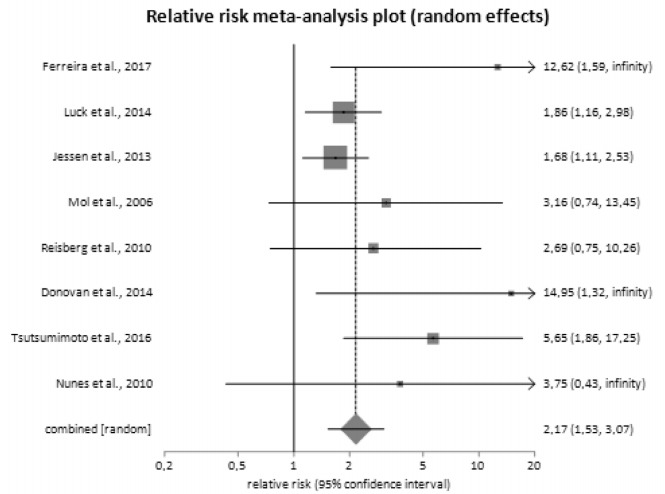
Relative risk of dementia.

Relative risk of dementia among patients with SCD compared to those without SCD was 2.17 (95%CI 1.53‒3.07; p<0.05) (Figure 3). There was heterogeneity (I^2^=11.20%; 95%CI 0.00‒61.00) and no evidence of bias (Harbord-Egger bias=1.51; p=0.01). After adjustment for age and gender, relative risk was 2.08 (95%CI 1.35‒2.89).

### Analysis of subjective cognitive decline progression to mild cognitive impairment

The following results were obtained from the analysis of 5 studies.

Cumulative conversion rate of SCD progression to MCI was 20.76% (95%CI 9.04‒35.73). Heterogeneity was high (I^2=^96.10%; 95%CI 93.90‒97.20) and there was some evidence of bias (Harbord bias=6.39; p=0.44). Annual conversion rate for MCI in the SCD group was 5.44% (95%CI 3.13‒8.33). Heterogeneity was high (I^2^=64.60%; 95%CI 0.00‒84.40).

Cumulative conversion rate for MCI in the control group was 8.93% (95%CI 6.84‒11.28). There was heterogeneity (I^2=^36.9%, 95%CI 0.00-75.90) and some evidence of bias (Harbord bias= -0.83; p=0.55). Annual conversion rate in the control group was 2.75% (95%CI 1.51‒4.34). Heterogeneity was high (I^2^=46.80%; 95%CI 0.00‒78.90).

**Figure 4.**
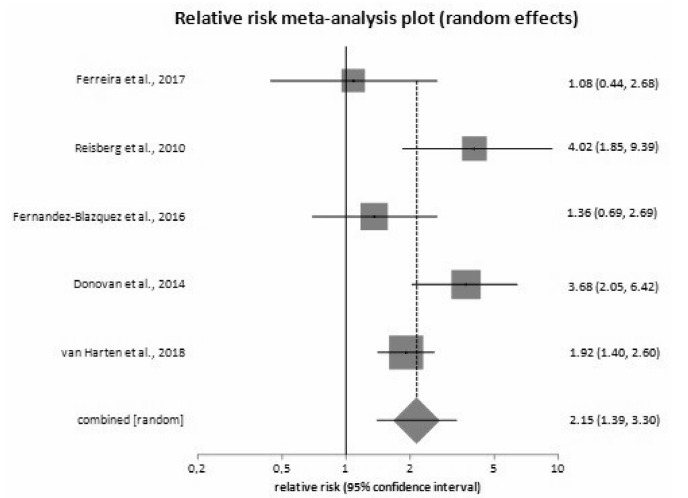
Relative risk of mild cognitive impairment conversion.

Relative risk of MCI conversion in SCD compared to control was 2.15 (95%CI 1.39‒3.30; p=0.005) (Figure 4). There was considerable heterogeneity (I^2=^58.80%; 95%CI 0.00‒82.60) and some evidence of bias (Harbord-Egger bias= -0.07; p=0.90). After adjustment for age and gender, relative risk was 2.12 (95%CI 1.10‒4.30).

**Figure 5.**
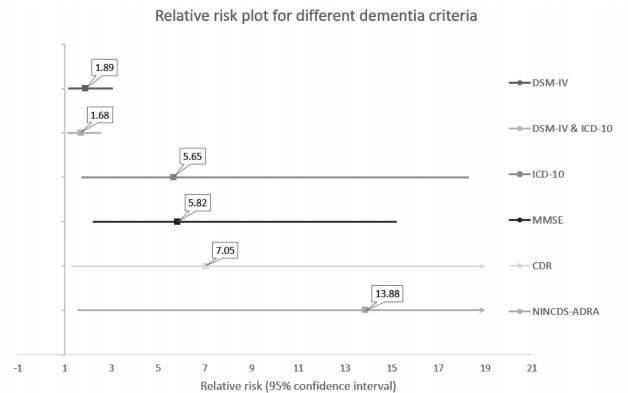
Relative risk plot for different dementia criteria.

DSM-IV: Diagnostic and Statistical Manual of Mental Disorders; ICD-10: International Statistical Classification of Diseases and Related Health Problems 10^th^ Revision; MMSE: Mini-Mental State Examination; CDR: clinical dementia rating; NINCDS-ADRA: Neurological and Communicative Disorders and Stroke and the Alzheimer’s Disease and Related Disorders Association Criteria.

The risk of dementia depended on the dementia criteria used. The highest relative risk of dementia was found in the study that used NINCDS-ADRA criteria and it was 13.88 (95%CI 1.59‒∞). The lowest relative risk was 1.68 (95%CI 1.11‒2.53) and it was found in the studies that used DSM-IV and ICD-10 criteria (Figure 5).

## DISCUSSION

The main aim of the review was to compare whether people with SCD are more likely to develop cognitive impairment over time than people without SCD. Cumulative risk of conversion to dementia in the SCD group is 7.23% (95%CI 3.64‒12.04). The relatively low risk of dementia conversion might be explained by the short duration of the follow-up period (5.27 years on average). The chance of progression to dementia in the SCD group is 2.17 (95%CI 1.53‒3.07; p<0.05) compared to normal aging.

The cumulative rate of SCD conversion to MCI was found to be 20.76% (95%CI 9.04‒35.73) over 4.91 years. The SCD group was 2.15 times more likely to progress to MCI than the group without SCD (95%CI 1.39‒3.30; p=0.005).

The results of our systematic review demonstrate that people with SCD are characterized by an increased risk of cognitive impairment.

The highest relative risk was found in the study that used NINCDS-ADRA criteria. We cannot offer an exact explanation of this finding. Perhaps, it might be due to the fact that these criteria were used in 1 study with a relatively small number of participants. We suppose that SCD precedes AD in most cases and NINCDS-ADRA criteria are used for AD diagnosis. The SCD and AD connection using NINCDS-ADRA criteria should be considered for further studies.

The assumption that SCD could precede cognitive impairment was confirmed by studies with biomarkers. It was found that individuals with SCD have an increased likelihood of AD-associated biomarker abnormalities.^8^ ^,^ ^25^ ^,^ ^26^ ^,^ ^27^ A study conducted by Visser et al. showed that SCD patients have AD-predicting CSF profile (low Aβ-42 and high tau levels) more often than control ones.^28^ These biomarkers are associated with a greater risk of MCI and dementia and, thus, SCD may expand indications for AD biochemical and bioimaging diagnostic screening.

Some SCD neuroimaging studies have been reported. Van der Flier et al. showed that individuals with SCD have a lesser left hippocampal volume than individuals without complaints.^29^ Another study found that SCD and amnestic MCI patients have similar MRI changes, including atrophy of the medial temporal and frontotemporal regions, correlating these findings with the severity of SCD.^30^ Several ﬂuorodeoxyglucose positron emission tomography (FDG-PET) studies identified hypometabolism in the parahippocampal gyrus, middle temporal gyrus, left inferior parietal lobe, inferior frontal gyrus, fusiform gyrus, thalamus, and in the right putamen^31^ ^,^ ^32^ in people with SCD.

SCD could be the earliest preclinical phase of dementing disorders in some patients. In particular, the beginning of dementia should be suspected in individuals who have memory complaints along with other dementia risk factors. However, the results of our study demonstrated that about 7% of people with SCD will have objective cognitive impairment in 5 years. This conversion rate is relatively low and there is no strong evidence that these patients should be treated as patients with cognitive impairment.

We did not investigate the relationship between SCD and depression, but we should note that several studies showed that individuals with higher depressive symptoms showed significant SCD-cognition association.^33^ ^,^ ^34^

Our study discovered that 2.75% of healthy subjects without SCD annually convert to MCI. These results should be considered along with the fact that MCI does not inevitably turn into dementia, but the reversion rate of MCI is high and ranges from 30 to 50% within two to five years of follow-up.^35^

One relevant issue is the lack of a standard definition of SCD and SCD criteria. Included studies used distinct SCD scales and assessment methods with different questions. Different cognitive complaints may affect the results of the study.

This study was not the first systematic review of SCD clinical data. A review performed by Mitchell et al. evaluated whether people with SCD are at increased risk of MCI and dementia.^36^ The authors included 32 studies, but there were some old studies that could impact the meta-analysis results due to misdiagnosis. In addition, not all the included studies had control groups, so the results of SCD groups were not compared to healthy controls. The annual conversion rate for MCI and dementia was slightly higher in comparison to our results. However, the relative risk of dementia conversion was 2.07. These results are close to our findings. Another meta-analysis performed by Burmester et al. was a large quantitative and qualitative synthesis of researches.^37^ However, the authors did not evaluate the annual conversion rate or relative risks of objective cognitive decline.

Our systematic review has some limitations. First, we had a relatively limited data set due to our strict inclusion criteria for the studies. Secondly, our review had heterogeneous data and some evidence of bias in the obtained results. High heterogeneity might be caused by different study settings and criteria used for the diagnosis. Evidence of bias can be explained by a relatively small number of included studies. Furthermore, some studies included a small number of participants.

Despite the limitations mentioned, the results of our systematic review demonstrate that patients with SCD have an increased risk of MCI or dementia. SCD is a risk group for MCI and dementia, and therefore worthy of further investigation and consideration for trials of new treatments.
